# Rush Medical College

**Published:** 1848

**Authors:** 


### ARTICLE V.

### RUSH MEDICAL COLLEGE.

The number of students in attendence at this Institution during the last session as shown by the catalogue was 142, double the number of any previous class.

This rapid increase in the number of its patrons from year to year affords ample evidence that the efforts of its faculty to offer every possible facility for the acquisicion of a thorough medical education are fully appreciated by the profession, and by students of the respective preceding classes.

In their behalf we can state that it is the intention of the faculty to comply with the recommendations of the medical association so far as to give a preliminary course of instruction during the month of October previous to the commencement of the regular term without additional charge. During this time the dissecting room, amply supplied with fresh material will be open to such as may wish to employ the time in the study of practical anatomy.

We are happy in being able to assure students also, that arrangements have been made for giving clinical instruction during the coming session such as will enable the teachers of Medicine, Surgery, and Obstetrics, to select from a large number of patients the most interesting cases and such as can be retained in the Hospital from the commencement to the termination of treatment, thus affording opportunities for acquiring knowledge of disease at the bed side superior even to those offered during the last term.

The hospital will be exclusively under the control of the faculty, and so arranged as to exclude cases without interest as well as all contagious diseases. H.

